# Predicting regional COVID-19 hospital admissions in Sweden using mobility data

**DOI:** 10.1038/s41598-021-03499-y

**Published:** 2021-12-17

**Authors:** Philip Gerlee, Julia Karlsson, Ingrid Fritzell, Thomas Brezicka, Armin Spreco, Toomas Timpka, Anna Jöud, Torbjörn Lundh

**Affiliations:** 1 Mathematical Sciences, Chalmers University of Technology, Gothenburg, Sweden; 2 Mathematical Sciences, University of Gothenburg, Gothenburg, Sweden; 3 Sahlgrenska University Hospital, Gothenburg, Sweden; 4 Department of Health, Medicine and Caring Sciences, Linköping University, Linköping, Sweden; 5 Center for Health Services Development, Region Östergötland, Linköping, Sweden; 6 Department of Laboratory Medicine, Faculty of Medicine, Lund University, Lund, Sweden; 7 Department of Research and Development, Skåne University Hospital, Lund, Sweden

**Keywords:** Viral infection, Applied mathematics, Epidemiology

## Abstract

The transmission of COVID-19 is dependent on social mixing, the basic rate of which varies with sociodemographic, cultural, and geographic factors. Alterations in social mixing and subsequent changes in transmission dynamics eventually affect hospital admissions. We employ these observations to model and predict regional hospital admissions in Sweden during the COVID-19 pandemic. We use an SEIR-model for each region in Sweden in which the social mixing is assumed to depend on mobility data from public transport utilisation and locations for mobile phone usage. The results show that the model could capture the timing of the first and beginning of the second wave of the pandemic 3 weeks in advance without any additional assumptions about seasonality. Further, we show that for two major regions of Sweden, models with public transport data outperform models using mobile phone usage. We conclude that a model based on routinely collected mobility data makes it possible to predict future hospital admissions for COVID-19 3 weeks in advance.

## Introduction

COVID-19 belongs to the category of infectious diseases that mainly are disseminated through transmission of the infectious agent in association with physical meetings (social mixing) between individuals. These meetings occur at home or at other locations such as workplaces or schools, which are reached using some means of transportation, e.g. by car, public transport or foot. The meetings tend to take on regular patterns and variations, and these can be used for different types of analytic purposes^1,2^.

The COVID-19 pandemic has affected the society in numerous ways. Before the availability of vaccines, the key intervention was the reduction in individual mobility, which was enforced either by strict legal lockdowns or, as in the case of Sweden, by recommendations to the general public. This enforced reduction in social mixing has had the intended effect of “flattening the curve” during the first waves of the pandemic^3^.

Obtaining an understanding of the effect of mobility-related social mixing on the transmission of COVID-19 requires an ability to measure and quantify said changes in individual mobility. This was achieved during the early phases of the COVID-19 pandemic by geographically tracking cell phone usage, either directly by mobile phone operators^4^ or via usage of Google services^5^ that are readily available for many geographic regions. In addition to this, mobility was also measured by considering the utilisation of public transport^6^.

This type of information has been used in a number of studies in order to model and understand the pandemic. Linka et al.^7^ used mobility data to obtain a correlation between the reproduction number and public health interventions, while Zhou et al.^8^ investigated the delay of outbreaks caused by mobility restrictions. Another application of mobility data is to make model-informed choices between different levels of social distancing^9^. Mobility data has also been used in order to draw conclusions about how markets and governments respond to surges in COVID-19 cases^10^, and to obtain a quantification of the impact on reduced mobility on COVID-19 cases and deaths^11^, and on the risk of transmission^12^.

Given the time delay between initial infection and potential hospital treatment, mobility data, such as records of daily commuters, also offer an opportunity to make predictions about the coming number of hospital admissions^13^. Such models can be useful for hospital administration since it allows for planning and a higher degree of preparedness for coming surges in the need of hospital beds. During the early phases of a pandemic there are limited possibilities to follow the dissemination of the infectious agent through laboratory testing. Therefore, knowledge of reliable syndromic data sources that can be used for response planning is essential during an emerging pandemic. The aim of this study was to investigate whether variations in data reflecting local social mixing levels through weekly commuting rates could be associated with later COVID-19 hospitalisation rates and also compare the ability of different data sources of commuting (or social mixing) to achieve this aim. The underlying assumption was that the levels of local commuting reflect the level of social mixing and correspondingly disease transmission and hospital admissions.

## Methods

A retrospective design was used for data collection and analysis. We developed an SEIR-model of disease transmission which outputs the expected number of hospital admissions. Here we describe the hospital admission and mobility data, the epidemiological model that we have used as well as the method for fitting the model to data. The code for the model and the data used is available at: https://github.com/philipgerlee/Predicting-regional-COVID-19-hospital-admissions-in-Sweden-using-mobility-data.

### Data

Data collected between week 10 (beginning 8th March) and week 50 (beginning with 7th December) of 2020 were used for the analyses.

#### Endpoint data

Weekly regional-level data on hospital admissions with the ICD-10 diagnosis code U07.1 ‘COVID-19, virus identified’^14^. The data are reported separately for each of the 21 regions in Sweden. Missing data points are replaced by zeroes for all regions.

#### Syndromic data

Mobility data from two sources were used: public transport data from the public transport authorities in two Swedish regions (Region Västra Götaland and Region Skåne) called Västtrafik (VT) and Skånetrafiken (ST), respectively, and Google mobility reports (GMR).

All of the above data was collected in accordance with the General Data Protection Regulation. These dataset are not considered personal data and therefore no approval from a licensing committee for collection was required. Consent to collect GMR was obtained by Google from their users.

The VT- and ST-data describe the total number of journeys made by public transport in the region and are reported on a weekly basis. Data are given in terms of a percent change compared to travel during week 9. The GMR-data also describes the change in mobility compared to a baseline, which is the median value from the 5-week period Jan 3-Feb 6, 2020. Mobility is split into place categories and we have used values from the category ‘transit stations’. The GMR-data is reported on a daily basis and in order to make it compatible with the model we calculate weekly averages. Figure S3 in the Supplementary Material shows the above mobility measures as a function of time.

### Epidemiological model

To model the weekly time series of COVID-19 related hospital admissions we have used an SEIR-model with time-dependent infectivity $\beta (t)$ which is informed by mobility measures. Infectivity is assumed to vary with mobility such that the number of new social contacts for each infected individual increases with travel.

We assume that mobility measured by public transport utilisation and mobile phone usage reflects the general level of social mixing in each region, which is then assumed to impact the contact rate and consequently the virus transmission. Note that we do not assume that disease transmissions occurs exclusively during travel, but rather that the above mobility measures serve as a useful proxy for the rate of new social contacts.

The model is defined in terms of the following set of coupled ordinary differential equations:

1 $$\begin{aligned} \left\{ \begin{array}{l} \dfrac{dS}{dt} = -\dfrac{\beta (t) SI}{N}\\ \dfrac{dE}{dt} = \dfrac{\beta (t) SI}{N} - \rho E\\ \dfrac{dI}{dt} = \rho E - \gamma I \\ \dfrac{dR}{dt} = \gamma I. \end{array} \right. \end{aligned}$$

Here $\rho$ is the rate at which people leave the exposed compartment, $\gamma$ is the rate of recovery and *N* is the population size of the region. In order to solve the system of equations we also need to specify an initial condition and when in time it occurs. We assume that all individuals are susceptible except an initial number of $I_0$ of infectious individuals at $t_0$ weeks prior to the first data point in the admission data (week 10).

To connect the dynamics of the SEIR-model with hospital admissions we assume that individuals in the infectious compartment give rise to future hospital admissions. To model this we assume that the number of hospital admissions $t_a$ weeks into the future is given by a fraction *p* of the present number of infectious individuals.

### Model parametrisation and fitting

The parameters of the SEIR-model were taken from previously published studies and we have used $\rho =1.37$ week$^{-1}$ (corresponding to a latency period of on average 5.1 days) and recovery rate $\gamma =1.4$ week$^{-1}$ (corresponding to a infectious period of on average 5 days)^15^.

Since testing was limited during the early stages of the pandemic in Sweden it is difficult to estimate the initial condition for our model. For simplicity we assume a single infected individual in a population of susceptibles appearing $t_0=4$ weeks prior to the first data point. We also tried to adjust the the initial condition for each region by assuming that a single infected individual was introduced 4 weeks prior to the first reported case in each region (obtained from^16^).

The scaling that relates the number of infected to hospital admissions was set to $p=0.023$ in accordance with a previous study^15^. The time lag from infection to hospital admission was set to $t_a = 3$ weeks. This value is related to the time from infection to hospital admission, which has been reported to be 17 days (5 days latency^15^ plus 12 days from symptom onset to admission^17^). However, it should not be interpreted as a parameter describing the fate of an individual patient, but should rather be interpreted as the time it takes for changes in disease transmission to propagate (sometimes via secondary cases) to hospital admissions. A previous study using mobility data has shown a time delay in admissions due to mobility restrictions in the range of 9–25 days^18^, which covers our assumed value of 21 days.

Given the uncertainty in many of the above parameter values we have carried out a sensitivity analysis by varying one parameter at a time within a reasonable range. The results of this analysis is presented in the Supplementary Material (Fig. S1).

The infectivity $\beta (t)$ is informed by the mobility data in the following way: For Västra Götaland and Skåne we use the public transport data and assume a linear relationship

$$\begin{aligned} \beta (t) = a+bV(t) \end{aligned}$$

where *a*, *b* are parameters that are fitted to the admission data (see below for details) and *V*(*t*) is the percent change in travel during week *t* compared to week 9. For all other regions we use the GMR-data in a similar way and assume that

$$\begin{aligned} \beta (t) = a+bG_i(t) \end{aligned}$$

where $G_i(t)$ is the GMR-data (place category ‘transit stations’) for region *i*, and *a*, *b* are parameters that are estimated.

In order to account for the fact that not only mobility changed at the onset of the pandemic, but also other circumstances such as physical distancing and increased hand hygiene, we adjust the baseline values for *V*(*t*) and $G_i(t)$ from 0 to 0.2.

The infectivity parameters *a*, *b* are estimated by minimising the mean squared error (RMSE)

$$\begin{aligned} E(\theta ) = \sqrt{\frac{1}{n}\sum _{i=0}^n \left( pI(t_i-t_a,\theta )-A(t_i) \right) ^2} \end{aligned}$$

with respect to $\theta =(a,b)$. Here $pI(t_i+t_a,\theta )$ is the predicted number of hospital admissions and $A(t_i)$ is the actual number of admissions and the sum runs over all time points $t_i$. To find the minimum RMSE we use a combination of global and local search. First we apply a global search using the grid search method with 80 linearly spaced values in the range 1-12 for both *a* and *b*^19^. The minimal value of the RMSE found in the grid search was subsequently used as an initial guess for the SciPy-function curvefit which implements the trust region reflective algorithm^20^. With this two-step minimisation of the RMSE we obtain for each Swedish region *i* a set ${\hat{\theta }}_i=({\hat{a}}_i,{\hat{b}}_i)$ of estimated parameters.

In order to quantify the uncertainty in our parameter estimates we calculated parametric bootstrap confidence intervals. This was achieved by independently drawing 1000 parameter combinations assuming normal distributions with means given by $({\hat{a}},{\hat{b}})$ and variances obtained from the trust region reflective algorithm. For each draw the numerical solution was calculated and confidence intervals were obtained by calculating the 2.5- and 97.5-percentile for each point in time.

When comparing the model error between different regions we normalise the RMSE by dividing by the maximum number of weekly admissions for each region.

In order to investigate correlations between regional variables (population size, population density and area) and the optimal parameters $({\hat{a}},{\hat{b}})$ and RMSE we performed a hypothesis test whose null hypothesis is that the slope of a linear regression between the variables is zero, using a Wald Test with t-distribution of the test statistic. The significance level was set to $\alpha = 0.05$ and we used a Bonferroni correction with $n=3$.

For Region Västra Götaland we fit the mobility-driven SEIR-model (1) using increasing amounts of reported hospital admissions. We start by including data up until week 20 and test the model’s predictive ability in terms of the mean absolute percentage error (MAPE) on the coming 3 weeks. This procedure is repeated for increasing amounts of training data. To illustrate the robustness of the model we also plot how the estimated model parameters ${\hat{a}}$ and ${\hat{b}}$ change as we include more weekly data.

## Results

### Predicting hospital admissions using public transport utilisation

**Figure 1:**
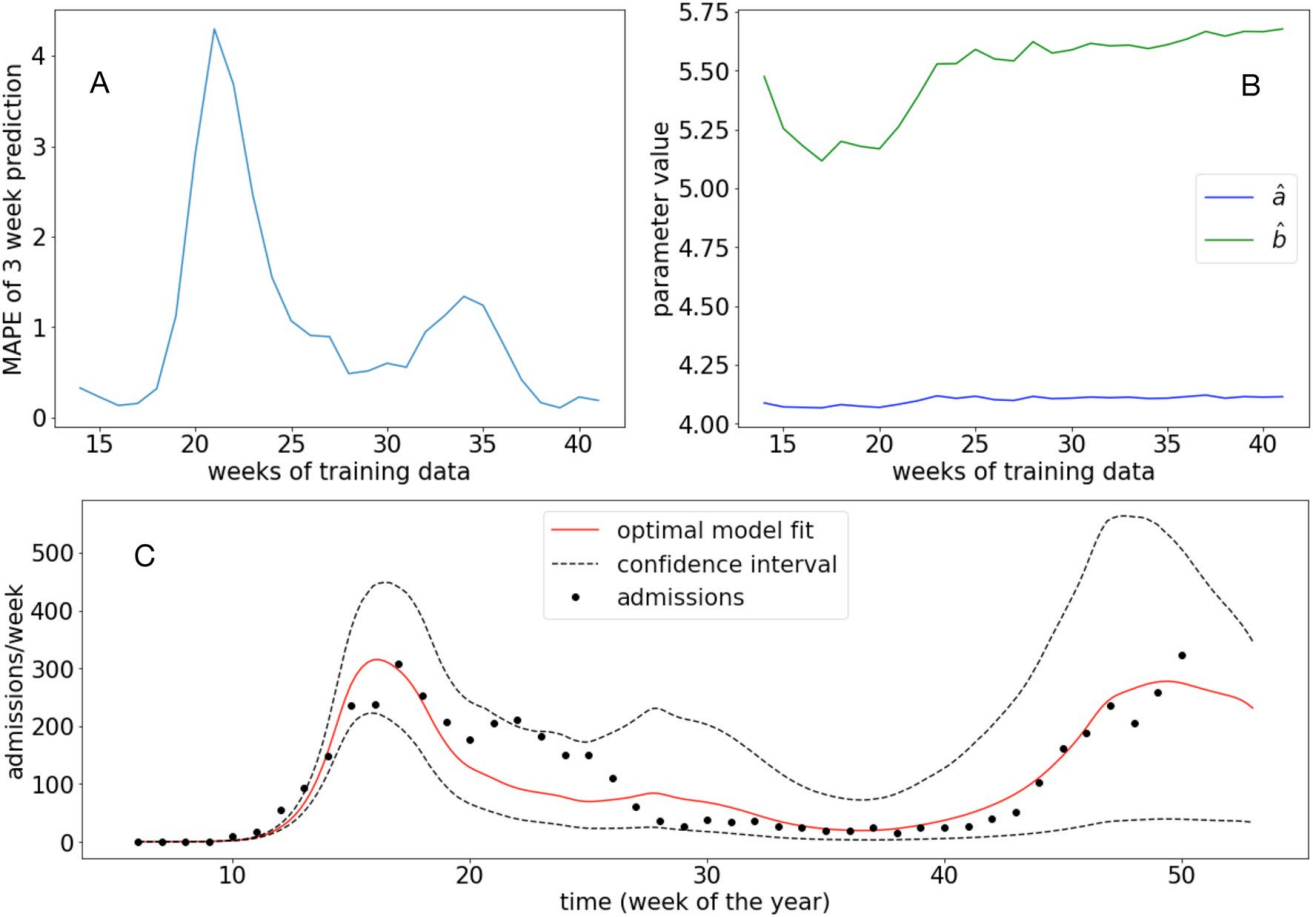
Model fit to admission data from Region Västra Götaland. (**A**) The model error in terms of the MAPE on 3 week predictions as a function of the number of weeks of data used in the fitting. (**B**) The estimated model parameters $({\hat{a}},{\hat{b}})$ as a function of the number of weeks of data used in the fitting. (**C**) The optimal fit when all data points are used (until week 45). The dashed lines show the 95% confidence interval for the model fit (see “Methods”).

For Region Västra Götaland the resulting model error in terms of MAPE can be seen in Fig. 1A. By successively increasing the training data, we see in Fig. 1B that the model remains largely unchanged beyond week 30, which timewise corresponds to the end of the first wave of the pandemic.

When using all available data we find that ${\hat{a}}=4.11$ and ${\hat{b}}=5.68$ (Fig. 1C), and we note that the model captures the dynamics of admissions during both the first and beginning of the second wave, although the rate of decline during the first wave is overestimated.

### Using Google mobility data to predict hospital admissions

**Figure 2:**
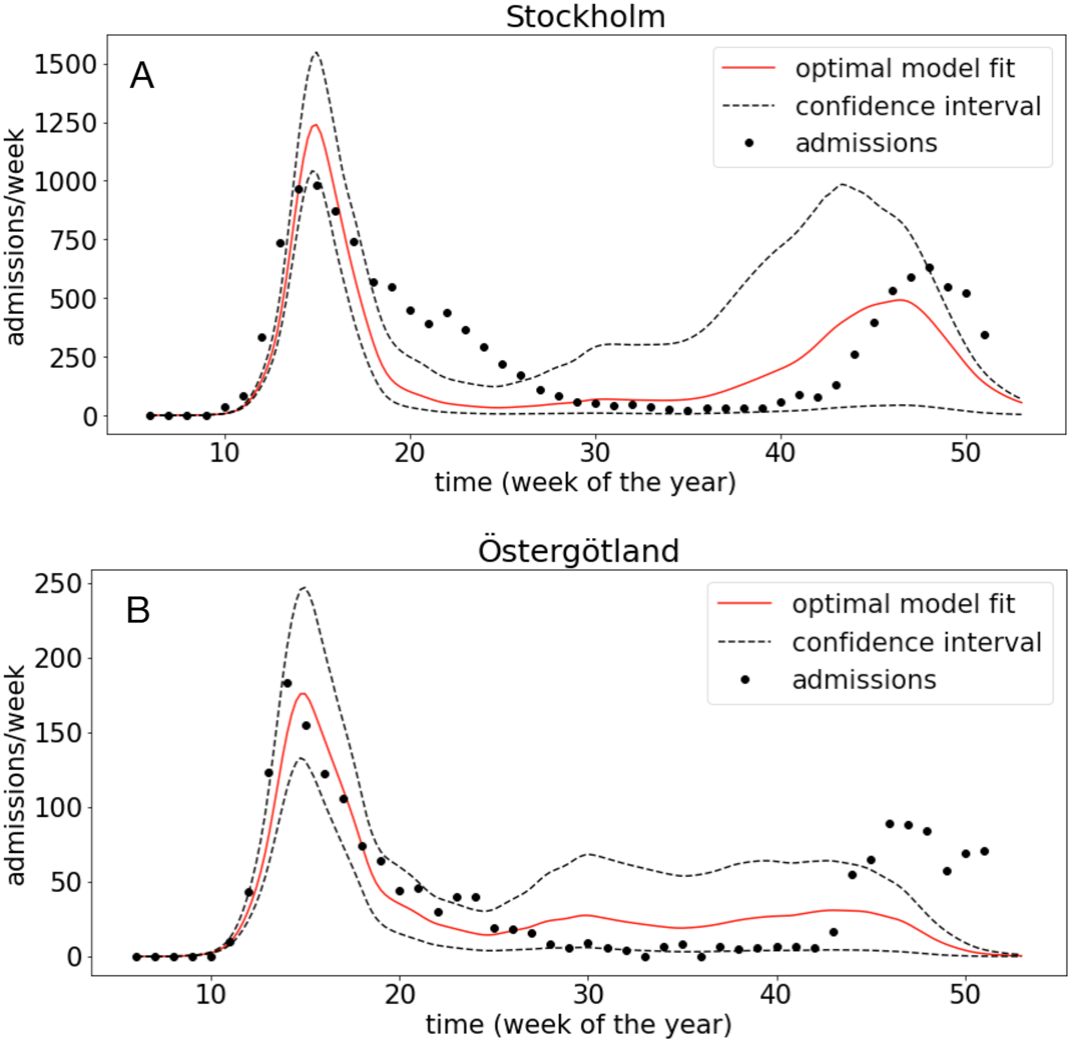
Optimal model fit for (**A**) Stockholm (${\hat{a}}=4.74$ and ${\hat{b}}=9.11$) and (**B**) Östergötland (${\hat{a}}=3.78$ and ${\hat{b}}=8.31$). In both panels the dashed lines show 95 % confidence intervals for the model fit.

For all other regions we make use of Google mobility data (see “Methods” for details). We tried to fit the model using both regionally adapted initial conditions (see “Methods”) and identical initial conditions for all regions (a single infected individual at week 6). The fit was not improved when using regional adaptation and we only present results for the uniform initial conditions. Figure 2 shows model fits for Östergötland and Stockholm (see Fig. S2 in the Supplementary Material for model fits to admissions in all Swedish regions and Table S1 for normalised RMSE and estimated parameters). Again, we note that the model correctly describes the timing of the first and second wave.

In order to investigate regional differences in model fit we compared the normalised RMSE and estimated parameter $({\hat{a}},{\hat{b}})$ to the population size, area and population density of each region. We found a significant correlation between ${\hat{a}}$ and regional population size (Pearsons’s correlation $\rho =0.73$) and population density ($\rho =0.73$), but no significant correlation between ${\hat{a}}$ and regional area. No such correlations were present between ${\hat{b}}$ and regional properties, nor between RMSE and regional properties.

### Public transport data improves model fit compared to Google mobility reports

**Figure 3:**
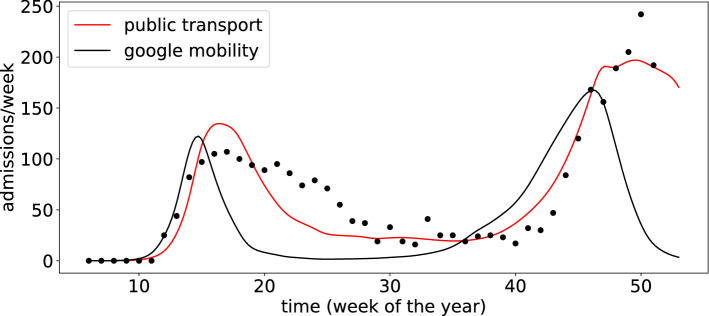
Optimal model fit for Skåne Region using mobility data from public transport (red line) and Google Mobility Report (black line). The model fit using public transport data is better compared to Google’s mobility data (RMSE of 21 vs. 62 admissions/week).

For Skåne Region we have both public transport data and GMR-data, which was used on all other regions. Figure 3 shows the best model fits using the mobility data from the public transport agency Skånetrafiken compared to GMR-data. We note that although the model using GMR fits the data during the second wave better the overall fit is considerably improved by using data from public transport. In terms of the RMSE we observe that the model error is 21 admissions/week for the public transport model compared to 62 admissions/week for the model that uses GMR. A similar trend is seen for Region Västra Götaland where public transport data yields an RMSE of 41 admissions/week whereas GMR-data gives an error of 118 admissions/week.

## Discussion

We set out to investigate whether variations in data reflecting local social mixing through weekly commuting rates were associated with later COVID-19 hospitalisation rates. We found that a SEIR-model can be fitted using two free parameters to regional data from Sweden and that COVID-19 hospital admissions can be predicted 3 weeks in advance using time-dependent mobility data.

Our approach is similar to that used by Chang et al.^19^ who used spatially resolved mobility data in order to model disease transmission in metropolitan areas in the US, and a study by Levin et al.^21^ who used data on county-to-county trips to fit an SEIR-model to COVID-19 spread in Minnesota. Both these studies compared their model output to COVID-19 incidence, whereas we have focused on hospital admissions. The reasons for this are twofold: firstly, the data on incidence in Sweden is unreliable due to variable testing strategies and secondly hospital admissions is a more interesting metric for healthcare providers.

Given the time lag between current mobility which drives infections and future hospital admissions, the model provides a tool for predicting the demands on hospital beds up to 3 weeks in advance. Although the SEIR-model describes the transmission of the disease, the model details were not in focus in the present study. Uncertainty in model parameters such as the initial condition and the fraction of individuals that become hospitalised implies that the model dynamics in terms of the number of susceptible, infectious and recovered individuals are unreliable. It is also worth pointing out that the connection between infection and hospitalisation is not assumed to be direct. It may well be that an individual who contributes to the measured mobility transmits the virus in several steps to an individual with an increased risk of severe illness who is subsequently hospitalised.

We have also assumed that the mobility measures we consider reflect the general level of social mixing. This fact can of course be questioned and it is possible that mobility data of the types considered in this study are biased in terms of socio-economic attributes. The observed reduction in mobility might overestimate the actual reduction in socio-economic groups where disease transmission exceeds that of the general population. In order to control for such bias one would have to collect other types of data that better reflect social mixing (e.g. using surveys as in the Polymod-study^22^) and compare it to public transport and GMR-data. This validation of mobility data has been carried out for mobile phone data in the US^23^.

Despite these simplifications, the model was able to capture the general shape and timing of both the first and the beginning of the second wave for most regions. A recurring feature seen across most regions is the inability of the model to accurately describe the width of the first peak (see e.g. Figs. 1C and 2A). The model tends to underestimate the actual width, and this is likely due to the lack of detail in the model or inaccuracy in the assumed parameter values. Moreover, it is noteworthy that the model captured the timing of the onset of the second peak without accounting for seasonal or temperature-driven infectivity. Instead, the results suggest that mobility in itself, which might contain seasonal variation is sufficient to capture the dynamics of hospital admissions. It should be noted that the separation into distinct waves can be made mathematically precise, which aids subsequent analysis e.g. in terms of mortality rates^24^.

There was no significant correlation between model error (in terms of normalised RMSE, see Table S1) and the population size, area or density of the regions. We found a significant correlation between the population size and density of a region and the estimated value ${\hat{a}}$. This parameter represent the baseline infectivity for each region, and assuming a constant probability of infection per contact across Sweden, regional differences in ${\hat{a}}$ reflect variation in the contact rate. Under this assumption the contact rate is correlated with population density, a phenomenon that has previously been reported for the COVID-19 pandemic in the US^25^. The reason for the correlation between regional population size and infectivity is probably due to the underlying correlation between population density and population size (Pearson’s correlation $\rho = 0.87$).

The present research has some limitations that should be taken in consideration when interpreting the results. When trained on admission data until week 20 for Region Västra Götaland the model initially performs well in terms of the model error (MAPE) on the 3 consecutive weeks (Fig. 1A). This is followed by an increase in the MAPE during week 18–22, which is due to an increase in admissions that the model is unable to capture, and then a subsequent decline. One possible explanation for this additional peak, which is also seen in other regions, is the Easter holidays that occurred during week 15. During the holidays people typically travel longer distances and meet family and friends, and these social contacts are captured by our mobility data to a lesser extent. In terms of the model robustness, we observe that the parameters remain largely unchanged after week 30, suggesting that data from the first wave was sufficient to fit the model (Fig. 1B).

In the model we have disregarded any kind of age-structure, migration of cases between regions and assumed a highly simplified connection between infection and hospital admission. In addition, we have assumed that the disease was introduced in an identical way in all regions. These choices were made in order to formulate a simple and general model, which could be applied directly to all regions. We have shown for Region Skåne that public transport data provides a better fit between model and admission data, and further tailoring the model to each region will most likely improve model fit even further.

The mobility measures we considered exhibit similar changes during the considered time period (see Fig. S3). Despite these similarities the public transport data provides a considerably better model fit compared to GMR. This suggests that public transport data is more complete and better reflects changes in social mixing at the regional level. If models for hospital admissions on a smaller spatial scale are to be constructed GMR might provide a better alternative due to its higher spatial resolution.

We have not considered data on the number of reported cases, which could also be used to estimate model parameters. The main reason for this is that during the time period we model (week 10–50, 2020), the testing strategy in Sweden has varied substantially. In addition, there are also regional differences in testing strategies. The delay from symptom onset to hospitalisation has been shown to be in the range of 3–10 days^26^, which means that the number of reported cases can be used to predict hospital admissions approximately a week ahead. In relation to this we note that our model provides predictions 3 weeks ahead.

In terms of numerical methods, we have also made a couple of simplifications. We carried out the parameter estimation one region at a time. Here it would be beneficial to consider a hierarchical mixed-effects model that considers all regions simultaneously^27^. We have performed a sensitivity analysis with respect to the initial condition, the parameters that relate the size of the infectious compartment to hospital admissions and the initial mobility (see Fig. S1). The results show that the model fit can be somewhat improved by making slight adjustment to the baseline parameter values. However, given the uncertainty in these parameter values we do not find it motivated to adjust our baseline values.

This study should be seen as a first attempt to model local hospital admissions in Europe early during an emerging pandemic using mobility data. The assumed 3-week lag between virus transmission and hospital admission implies that the model can, using routinely available mobility data, make predictions 3 weeks into the future. The results encourage continued research on use of mobility data, which is easily accessed, in health service capacity planning during the early phases of a pandemic when the availability of laboratory testing resources are limited.

## Supplementary Information

Supplementary Information.
