# Supplementary material for: Predicting regional COVID-19 hospital admissions in Sweden using mobility data
Source: Sci Rep. 2021 Dec 17;11:24171. doi: 10.1038/s41598-021-03499-y (PMC8683437; doi:10.1038/s41598-021-03499-y)
Supplement: Supplementary file 1 — Supplementary Information. [file 41598_2021_3499_MOESM1_ESM.pdf]

I. SUPPLEMENTARY MATERIAL

A. Sensitivity analysis

Figure S1 shows how the model error (RMSE) of the best fit for Region Västra Götaland changes when the parameters p, t_a,I_0 and $V(t=0)$ are varied. We note that it is possible to achieve a slightly better model fit when the probability of hospitalisation is lowered to $p = 0.1$, but the improvement in model fit is minor. For the delay we see that our value of $t_a = 3$ weeks lies close to a local minimum, but little would be gained (in terms of RMSE) by increasing the delay. The number of infected individuals at $t = 0$ has a more complicated impact on the error. A smaller RMSE could be achieved by increasing I_0 from its default value of 1, but the improvement is again minor. Lastly, the initial infectivity has a minor impact on the model error as long as it remains below 0.6.

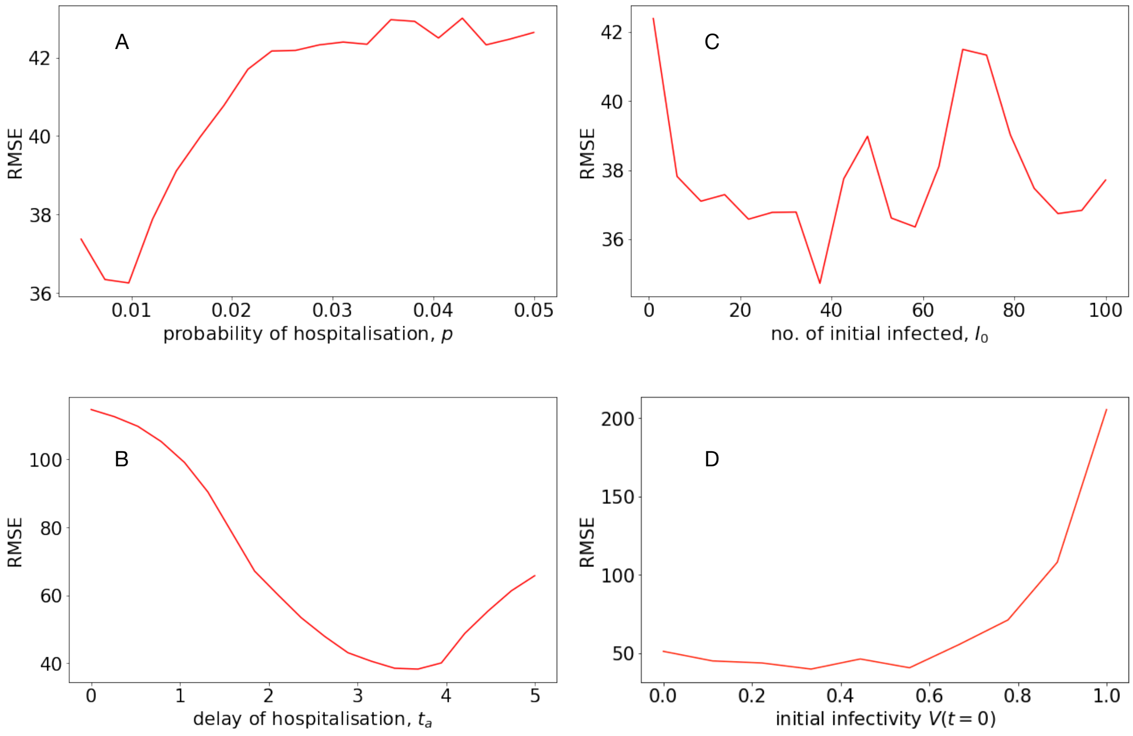

FIG. S1. Sensitivity analysis of model parameters for Region Västra Götaland. The defauly values are $p = 0.023$, $t_a = 3$, $I_0 = 1$ and $V(t = 0) = 0.2$.

B. Fitting the model to 20 Swedish regions

Here we present model fits for all Swedish regions expect Gotland for which no data was available from the National Board of Health and Welfare.

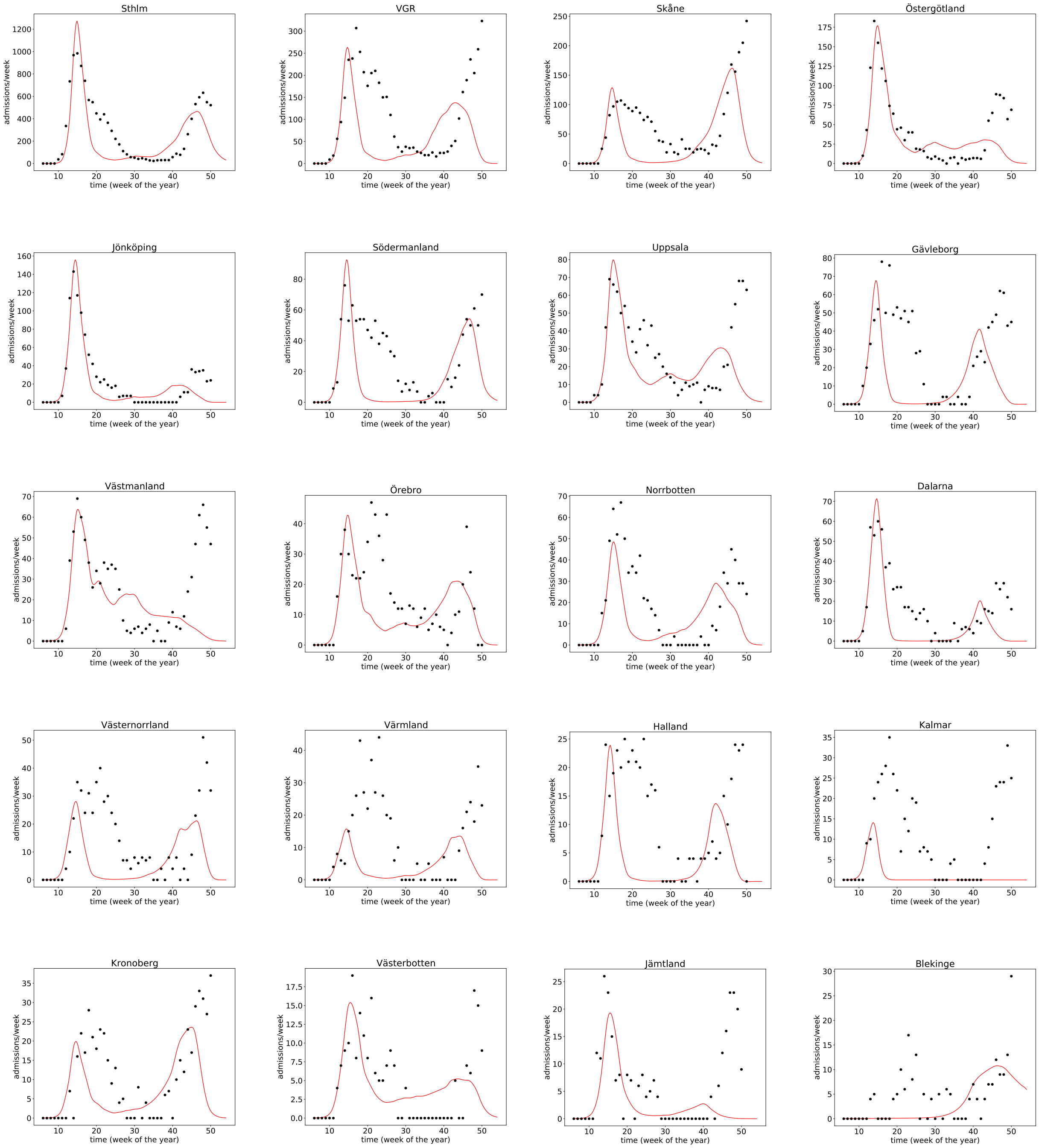

FIG. S2. Optimal model fit for all Swedish regions except Gotland. Estimated parameter values can be found in table S1.

Region	Population size (N)	Area (km^2)	$\hat{a}$	$\hat{b}$	normalised RMSE
Stockholm	2389923	6524	4.752653	8.985473	0.185063
Västra Götaland	1725881	23800	3.879982	9.530924	0.337702
Skåne	1387650	10968	3.662573	8.139158	0.236140
Östergötland	467095	10559	3.782899	8.322228	0.149207
Jönköping	364750	8190	3.445867	10.879671	0.106939
Södermanland	299101	10437	3.544680	8.425909	0.313578
Uppsala	387628	5427	3.577442	6.566021	0.304878
Gävleborg	287660	8504	3.123226	10.237733	0.367708
Västmanland	277074	6075	3.506538	6.693333	0.290102
Örebro	305726	28029	3.248453	7.747718	0.295193
Norrbotten	249768	18118	3.186880	7.267795	0.257602
Dalarna	287806	17519	2.977896	10.652902	0.229170
Västernorrland	244855	5118	2.937819	9.170180	0.325113
Värmland	282840	54665	2.729904	8.900794	0.362670
Halland	336132	97239	2.620944	10.085443	0.461986
Kalmar	245992	11165	2.380262	11.197420	0.405193
Kronoberg	202163	21549	2.997906	6.992810	0.303907
Västerbotten	273061	8424	2.966066	5.573555	0.242103
Jämtland	130972	2931	2.881223	5.408800	0.290599
Blekinge	159349	48935	1.729996	1.387774	0.194318

TABLE S1. Population size, area, estimated parameters and model error (normalised RMSE) for all considered regions.

C. Mobility data for Västra Götaland

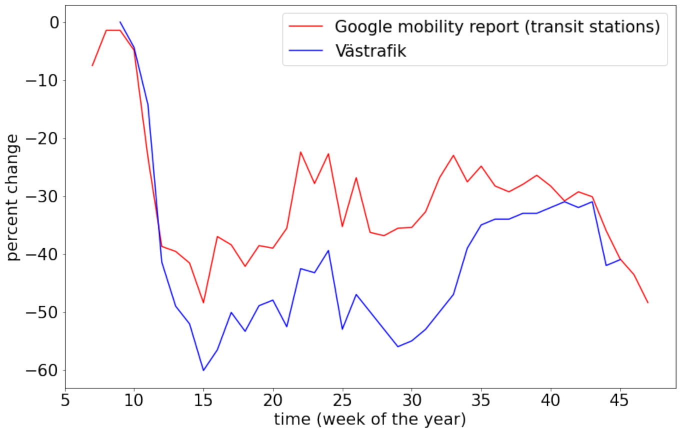

FIG. S3. Mobility data for Region Västra Götaland in terms of public transport usage (blue) and Google mobility report (red). See methods for details.
